# Probable Vogt–Koyanagi–Harada Disease after COVID-19 Vaccination: Case Report and Literature Review

**DOI:** 10.3390/vaccines10050783

**Published:** 2022-05-16

**Authors:** Xinyi Ding, Qing Chang

**Affiliations:** 1 Department of Ophthalmology, Eye and ENT Hospital of Fudan University, Shanghai 200031, China; dr_xding@outlook.com; 2 Shanghai Key Laboratory of Visual Impairment and Restoration, Fudan University, Shanghai 200031, China; 3 Key Laboratory of Myopia of National Health Commission, Fudan University, Shanghai 200031, China; 4 Key Laboratory of Myopia, Chinese Academy of Medical Sciences, Shanghai 200031, China

**Keywords:** COVID-19, vaccination, adverse events, ocular complications, Vogt–Koyanagi–Harada disease

## Abstract

COVID-19 vaccination is considered the most effective and promising approach for the elimination of the SARS-CoV-2 pandemic globally. Although the vaccine has been proven to be safe, as evidenced by the promotion of mass vaccination, new side effects, including several ocular complications that were not described during the experimental stage, are now emerging. In the present study, we report a 33-year-old Chinese man who developed probable Vogt–Koyanagi–Harada (VKH) disease only one day after his first dose of an inactivated COVID-19 vaccine, without any systemic symptoms. His medical history was unremarkable, except for hypertension. Although successfully relieved by oral prednisone, the patient progressed to the chronic stage of VKH disease with ocular depigmentation 4 months after onset. By reviewing similar cases previously reported, we discuss and summarize the common characteristics of VKH disease associated with vaccines against SARS-CoV-2, as well as the possible mechanisms behind this phenomenon. Although the causality is unclear, ophthalmologists and generalists should be aware of this possible ocular adverse effect after COVID-19 vaccination.

## 1. Introduction

Vaccination against COVID-19 appears to be an essential approach to control the pandemic, leading not only to a consistent reduction in the risk of SARS-CoV-2 infection, but also preventing severe clinical manifestations in infected people [1]. Although robust data obtained from the general population have confirmed the safety of diversified SARS-CoV-2 vaccines [2,3,4], unexpected medical events in temporal association with vaccination occur continuously. In particular, ocular complications following COVID-19 vaccination are major concerns, although the causality is still a matter of debate. To date, several ocular adverse effects have been reported, including corneal graft rejection, Bell’s palsy, retinal venous occlusion, retinal artery occlusion, acute macular neuroretinopathy, central serous chorioretinopathy and a variety of uveitis syndromes, etc. [5,6]. Vogt–Koyanagi–Harada disease (VKH) is a multisystem granulomatous inflammatory disorder of unknown cause that is characterized by bilateral panuveitis, as well as auditory, neurological, and integumentary manifestations in its complete form [7]. Here, we report a patient who presented with probable VKH disease in close temporal association with an inactivated COVID-19 vaccine.

## 2. Case Report

Four days after receiving his first dose of an inactivated COVID-19 vaccine (Sinopharm (Vero cell), inactivated COVID-19 vaccine) in early June 2021, a 33-year-old Chinese man presented at our hospital with a three-day history of vision decline and metamorphopsia in both of his eyes, without tinnitus, an intense headache or any other systemic symptoms. His wider medical history was unremarkable, except for hypertension found a year ago, which was 140/90 mmHg during the physical examination. He had no history of penetrating ocular trauma or surgery preceding the initial onset of uveitis.

**Figure 1:**
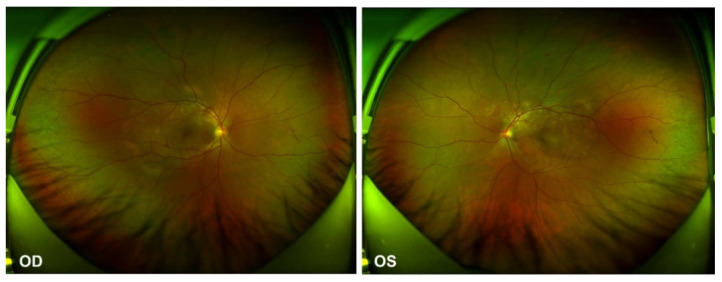
Ultra-widefield Scanning Laser Ophthalmoscope (SLO) retinal imaging during the first evaluation: multifocal serous retinal detachment OU.

**Figure 2:**
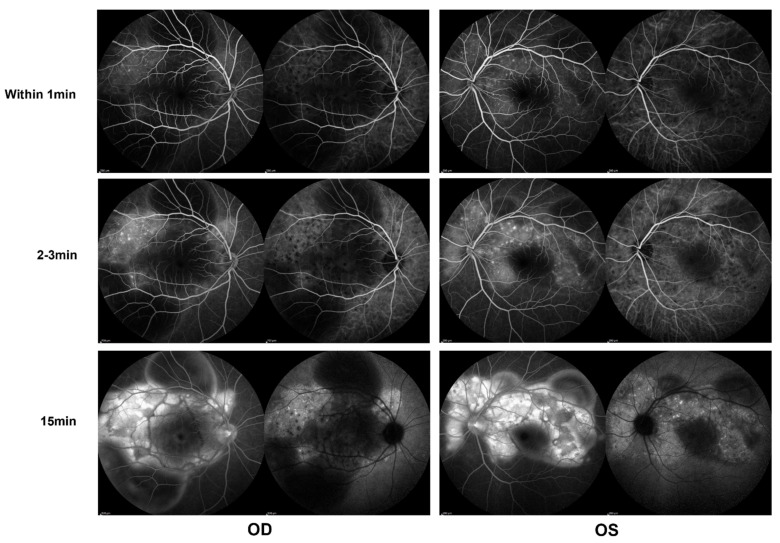
Indocyanine green angiography (ICGA) and fluorescein angiography (FA) (Heidelberg) during the first evaluation, showing early choroidal perfusion inhomogeneity, dot hyperfluorescence, and multiple areas of pinpoint hyperfluorescent foci with later-phase pooling OU.

**Figure 3:**
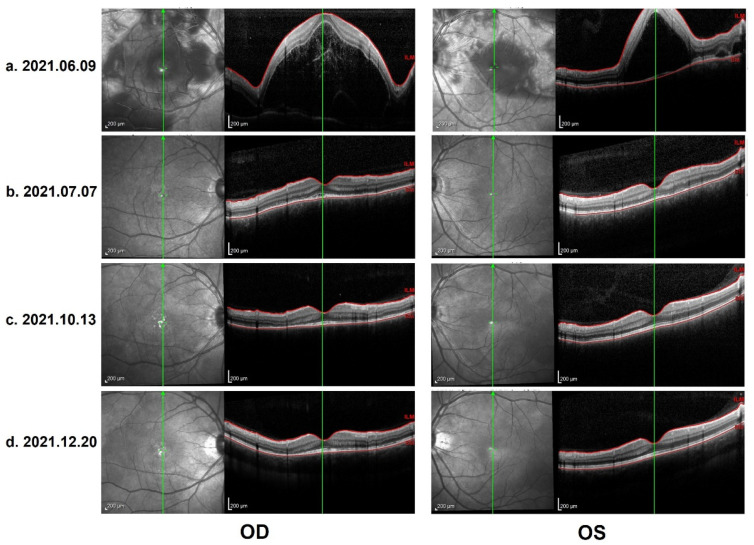
(**a**) OCT on 9 June 2021: Heidelberg optical coherence tomography (OCT): bullous serous retinal detachment, retinal pigment epithelium (RPE) undulation and membranous structure on the RPE beneath the foveal cystoid space. (**b**) Control OCT on 7 July 2021: total resolution of subretinal fluid, discontinuous inner segment/outer segment (IS/OS) layer and rough RPE. (**c**) Control OCT on 13 October 2021: further improvement in IS/OS layer and RPE, with a melanin change in the choroid stroma. (**d**) Control OCT on 20 December 2021 (the last evaluation): stable and similar to the previous follow-up.

In the ophthalmological evaluation, his best-corrected visual acuity was 20/400 in OD and 20/70 in OS. Examination under a slit lamp revealed a quiet anterior segment in both eyes, without keratic precipitates or flare in the anterior chamber. Dilated fundus examination revealed multifocal serous retinal detachment OU without vitritis, captured by an ultra-widefield Scanning Laser Ophthalmoscope (SLO) retinal imaging system (Optos 200Tx, Optos PLC, Dunfermline, UK) (Figure 1). Indocyanine green angiography (ICGA) and fluorescein angiography (FA) (Heidelberg Engineering, Heidelberg, Germany) revealed early choroidal perfusion inhomogeneity, dot hyperfluorescence, and multiple areas of pinpoint hyperfluorescent foci with later-phase pooling OU (Figure 2). Optical coherence tomography (Spectralis OCT; Heidelberg Engineering, Heidelberg, Germany) and enhanced-depth imaging swept-source optical coherence tomography (EDI SS-OCT, PLEX Elite 9000^®^device, Carl Zeiss Meditec, Dublin, CA, USA) confirmed a series of morphological changes OU (bullous serous retinal detachment, retinal pigment epithelium (RPE) undulation, significant choroidal thickening and the development of a membranous structure on the RPE beneath the foveal cystoid space (Figure 3a). Widefield swept-source optical coherence tomography angiography (SS-OCTA, PLEX Elite 9000^®^device, Carl Zeiss Meditec, Dublin, CA) with a 12 × 12 mm montage exhibited a distributed flow void, which was not limited to the posterior pole in the choriocapillaris layer OU. Blood work, including a test for human immunodeficiency virus (HIV), a Treponema pallidum particle assay (TPPA), a rapid plasma reagin (RPR) test and a test for autoinflammation markers, was unremarkable.

**Figure 4:**
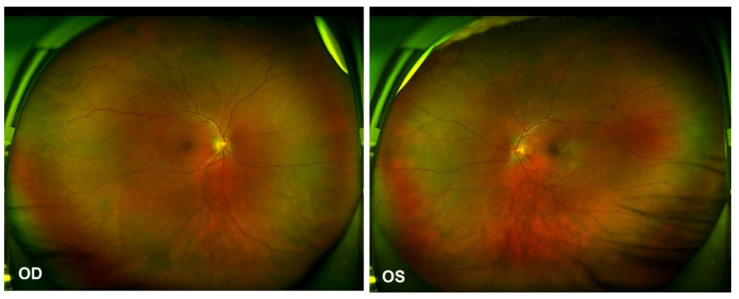
Ultra-widefield Scanning Laser Ophthalmoscope (SLO) retinal imaging on 13 October 2021: multifocal serous retinal detachment OU.

As per the revised diagnostic criteria for VKH disease established at the First International Workshop [7], our patient was diagnosed with probable Vogt–Koyanagi–Harada disease, without any neurological, auditory or integumentary findings. In addition, vaccination against SARS-CoV-2 could have been an immunological trigger for VKH disease in this patient, considering that there was a close temporal association between COVID-19 vaccination and the initial presentation of VKH disease. Therapy with 60 mg per day of oral prednisone was provided initially. Two weeks after the first evaluation, the patient reported a significant improvement in his visual acuity OU, which was 20/40 in the OD and 20/25 in the OS. The control OCT images showed a total resolution of subretinal fluid, a discontinuous inner segment/outer segment (IS/OS) layer and a rough RPE (Figure 3b). The prednisone dosage was tapered gradually. Four months later, further improvement in the IS/OS layer and RPE was revealed and the visual acuity returned to 20/20 OU. However, the patient presented with a “sunset glow” fundus OU from that point (Figure 4), with the melanin change in choroid stroma captured by OCT (Figure 3c,d). As there were no signs of inflammation relapse, no additional treatments were administered, except for oral prednisone.

The renal function of the patient was found to be abnormal one month after the first evaluation, with elevated serum creatinine (154 umol/L) and urea nitrogen (9.1 mmol/L). A kidney biopsy was then performed, and stage III chronic kidney disease (CKD) (IgA nephropathy, nephrosclerosis (70%), hypertensive nephropathy) was diagnosed. Amlodipine (5 mg qd), bisoprolol (1.25 mg qd), and sodium hydrogen carbonate (NaCHO3) (500 mg qd) were provided to normalize the blood pressure and maintain the acid–alkali equilibrium.

A score of four was calculated using the Naranjo adverse drug reaction probability scale (Table 1), which indicates that the symptoms observed were a possible adverse drug reaction [8].

## 3. Discussion

VKH disease is a multisystem disorder characterized by bilateral granulomatous panuveitis, which is frequently associated with neurological, auditory, and integumentary manifestations. It is typically classified into four consecutive stages with different clinical manifestations: the prodromal stage, the acute stage, the chronic convalescent stage and the chronic recurrent stage [7]. The etiology and pathogenesis of VKH disease still require further exploration.

Although an innate immune response against melanin and melanocytes in multiple organs is the most accepted theory in the etiology of VKH disease so far, VKH disease is widely believed to have some triggering factors, such as genetic susceptibility and microbial infection [9,10]. Epstein–Barr virus (EBV) Deoxyribonucleic acid (DNA) has previously been detected in the vitreous of a patient with VKH disease [11]. However, EBV is a ubiquitous virus in humans and a case report could not be regarded as strong evidence of its role in VKH disease. What is more convincing is that B lymphocytes established from patients with VKH were proven to contain greater amounts of EBV DNA and be more susceptible to EBV activation [12]. Further studies indicated that the seroprevalence of cytomegalovirus (CMV) was significantly higher in patients with VKH, and there was cross-reaction of peptide-specific T cells with CMV-egH and tyrosinase, suggesting that CMV might be responsible for the onset of VKH disease [13]. Cases of VKH disease associated with microbial infection, such as influenza A virus, mycoplasma pneumoniae, and tuberculosis, have been reported in the literature [14,15,16]. With the pandemic of SARS-CoV-2 in recent years, COVID-19 infection has also been reported as a stimulator of VKH disease [17,18]. It is worth noting that the intense relationship between VKH disease and diversified vaccines has been more commonly recorded, including for vaccines against Bacillus Calmette-Guerin (BCG), yellow fever, hepatitis B, papillomavirus, and COVID-19 [16,19,20,21,22].

Saraceno et al. described a case of Vogt–Koyanagi–Harada disease following the administration of the ChAdOx1 nCoV-19 (AZD1222) vaccine in 2021 for the first time [19]. In this case, a 62-year-old healthy female patient developed a severe headache and tinnitus 2 days after receiving the Oxford-AstraZeneca Chimpanzee Adenovirus Vectored Vaccine ChAdOx1 nCoV-19 (AZD1222). Two days later, she developed an acute loss of vision in both eyes. Her BCVA was 20/600 in OD and 20/200 in OS. Mild inflammation in the anterior chamber, serous retinal detachment and optic disc hyperemia OU were observed. OCT showed bilateral serous retinal detachment, bacillary layer detachment and subretinal hyperrefective dots. She was treated with oral systemic prednisone (1.5 mg/kg/day). After 3 weeks, she presented with a BCVA of 20/20 OU, no signs of inflammatory activity, and no retinal detachment.

Table 2 displays the clinical characteristics of our case in comparison with the other previously reported cases of VKH disease or patients with VKH-like symptoms in temporal association with COVID-19 vaccination. Twelve case reports were listed, while case series associated with ocular adverse events, including VKH disease or VKH-like symptoms, were not included, due to incomplete clinical data. Out of the 12 cases, there were 3 males and 9 females, with an average age of 48.92 ± 13.60 (range 19–72 years old). Two patients experienced recurrent VKH disease, while ten patients were diagnosed with newly onset VKH disease or VKH-like symptoms (four with probable VKH disease, two with incomplete VKH disease, one with complete VKH disease and three with VKH-like symptoms). The onset of new or exacerbated symptoms following the first or second vaccination (including with adenovirus vectored vaccines, mRNA vaccines and inactivated vaccines) ranged from 12 h to 3 weeks. Three out of twelve patients suffered vision decline twice following two vaccine shots at short intervals. Vision acuity ranged from hand motion to 20/20, according to the severity and course of the choroiditis. With prompt treatment, visual acuity in 9 patients recovered to or stayed stable at 20/20 OU, while VA in other patients was documented to have improved well, although it had not recovered back to normal. Sunset glow fundus was exhibited in one patient diagnosed with complete VKH disease with poliosis. Oral steroids were the fundamental treatment chosen in 11 out of 12 cases. One case of VKH-like symptoms with mild manifestations recovered merely with several shots of periocular triamcinolone acetonide. Intravenous steroids were used in five cases as part of the initial pulse therapy, with the use of an immunosuppressant in one case and biological agent (infliximab) in another.

The above literature review demonstrates several features of VKH disease or VKH-like symptoms associated with COVID-19 vaccination. Firstly, both the initial and subsequent shots of the COVID-19 vaccine have been reported to trigger VKH disease or VKH-like symptoms. Secondly, the three types of vaccines most widely used are considered to be probable initiating factors of autoimmunity, presented as VKH disease or VKH-like symptoms. Thirdly, most occurrences of newly onset, exacerbated and recurrent VKH disease or VKH-like symptoms were within the time window of increased risk after a plausible cause (within 40 days) described in the specific guidelines (Causality Assessment of an Adverse Event Following Immunization—AEFI) [23]. Otherwise, integumentary manifestations are not common, nor are chorioretinal depigmented presentations, such as sunset glow fundus. Finally, although there were different levels of manifestations and visual damage during the first evaluation, there was a good response to steroid therapy and an ideal visual prognosis following COVID-19 vaccination.

The precise mechanisms behind this phenomenon are unclear. Molecular mimicry, defined as “the similarity between a host epitope and an epitope of a microorganism or environmental agent”, has been seen as one of the most likely processes associated with these innate immune responses following vaccination, such as VKH disease [24]. Sachinidis and Garyfallos have recently addressed a possible explanation for these immunity reactions, emphasizing the role of age-associated B cells [25]. Teijaro et al. suggest that vaccine-induced autoimmune reactions are mainly based on the agonists of the Toll-like receptor (TLR)-7/8 or TLR-9. The latter is known to play a major role in the pathogenesis of autoimmune diseases [26,27]. Talotta et al. and Akinosoglou et al. propose that vaccination does not generate new autoimmune diseases, but rather triggers long-lasting latent autoimmunity [28,29], which emphasizes the importance of evaluating profile risk prior to vaccine administration.

Furthermore, we need to consider the possible causal link between kidney damage and recent vaccination. Except for the numbers of case reports, a series of 29 cases of glomerular disease, including IgA nephropathy, minimally changed disease, membranous nephropathy, and Anti-neutrophil cytoplasmic antibodies (ANCA)-associated Glomerular Nephritis (GN), suggested that newly onset or recurrent glomerular disease should be monitored as a potential adverse event of the COVID-19 vaccine [30]. In our case report, although the IgA nephropathy discovered one month after the patient’s first evaluation was considered to exist long before the onset of VKH disease, according to the clinical (stage III CKD) and pathological (nephrosclerosis (70%)) diagnosis, we cannot rule out the possibility that the vaccine aggravated the kidney damage.

**Table 2:** Clinical characteristics of our case in comparison with the other previously reported patients with Vogt–Koyanagi–Harada (VKH) or VKH-like symptoms in temporal association with the COVID-19 vaccination [19,24,31,32,33,34,35,36,37,38,39,40].

Study	Age/Sex	Eye	Medical history	VA	Symptoms	Onset of Symptoms	Diagnosis	Vaccine Dose	Vaccine	Immunusuppressive Treatment	Recovery
Current case	33/M	OU	Hypertension	20/400 (OD) 20/70 (OS)	Bilateral blurred vision	1d	Probable VKH	first	Inactivated vaccine	Oral steroids	20/20 OU (4 months later)
Bilateral Panuveitis Mimicking Vogt-Koyanagi-Harada Disease following the First Dose of ChAdOx1 nCoV-19 Vaccine	72/F	OU	Cataract surgery	20/40 (OU)	Bilateral blurred vision, headache, neck stiffness, and tinnitus	3d	VKH-like	first	Adenovirus vectored vaccine	0.5% loteprednol etabonate, intravenous and oral steroids	20/20 OU (6 weeks later)
Acute-onset Vogt-Koyanagi-Harada-like uveitis following COVID-19 inactivated virus vaccination	19/M	OU	No	20/20 (OU)	Bilateral eye redness and blurred vision	12h	VKH-like	first	Inactivated vaccine	Periocular injections of triamcinolone acetonide	Blurred vision resolved in five days without recurrent through four months of follow-up
Harada-like syndrome post-Covishield vaccination: A rare adverse effect	30/F	OU	No	Finger counting 1 m (OD) Finger counting in front of the face (OS)	Bilateral blurred vision	2d (First) 1d(Second)	Probable VKH-recurrent	first & second	Adenovirus vectored vaccine	Oral steroids	Good improvement currently
Vogt-Koyanagi-Harada Disease Exacerbation Associated with COVID-19 Vaccine	46/F	OU	LASIK	20/32 (OU)	Bilateral blurred vision and headaches after the first dose, photophobia and worsening of visual acuity and dysacusis impairment	2d (First) 4d (Second)	Incomplete VKH-complete VKH	first & second	mRNA vaccine	Intravenous and oral steroids	20/20 OU (2 weeks later)
Vogt-Koyanagi-Harada Relapse after COVID-19 Vaccination	54/F	OD	VKH	20/600 (OD) 20/20 (OS)	Central scotoma OD	9d	Recurrent VKH	first	mRNA vaccine	Periocular injection of triamcinolone acetonide, intravenous and oral steriods, AZA	20/20 OU (5 months later)
De Novo Vogt-Koyanagi-Harada Disease following COVID-19 Vaccine: A Case Report and Literature Overview	57/F	OU	No	20/65 (OD) 20/50 (OS)	Bilateral blurred vision and headaches	3w	Incomplete VKH	first	mRNA vaccine	Mydriatic drops and peribulbar injections of dexamethasone, intravenous and oral steroids	20/20 OU (3 months later)
Vogt-Koyanagi-Harada Disease Associated with COVID-19 mRNA Vaccine	54/M	OU	Type 2 Diabetes Mellitus and hyperlipidemia	20/600 (OD) 20/20 (OS)	Acute onset bilateral, sequential blurring of vision	1d	Probable VKH	first	mRNA vaccine	Intravenous and oral steroids	10/20 OU (13 days)
Reactivation of Vogt-Koyanagi-Harada disease under control for more than 6 years, following anti-SARS-CoV-2 vaccination	43/F	OU	Imcomplete VKH	20/20 (OU)	Nothing special	6w	Recurrent VKH	Second	mRNA vaccine	Oral steroids and Infliximab	20/20 OU
Bilateral uveitis after inoculation with COVID-19 vaccine: A case report	50/F	OU	No	20/33 (OD) 20/66 (OS)	Bilateral blurred vision and visual distortion	5d	Probable VKH	first	Inactivated vaccine	Periocular injection of triamcinolone acetonide, oral steriods	20/25 (OD) 20/20 (OS) (5 weeks)
Vogt-Koyanagi-Harada Syndrome following COVID-19 and ChAdOx1 nCoV-19 (AZD1222) vaccine	62/F	OU	No	20/600 (OD) 20/200 (OS)	Acute loss of vision	4d	Incomplete VKH	second	Adenovirus vectored vaccine	Oral steroids	20/20 OU (3 weeks)
Panuveitis following vaccination for COVID-19	43/F	OU	No	20/500 (OU)	Substantial vision loss, eye pain, eye redness, and sensitivity to light.	3d	VKH-like	second	mRNA vaccine	Difluprednate and cycloplegic drops, oral steroids	20/20 OU (3 weeks)
Ocular Adverse Events after Inactivated COVID-19 Vaccination in Xiamen	57/F	OU	No	Hand motion (OD) 20/80 (OS)	Severe visual loss	10d (First) 2d (Second)	Probable VKH-exacerbation	first & second	Inactivated vaccine	Oral steriods	20/20 OD (4 weeks)

## 4. Conclusions

VKH disease is a form of bilateral granulomatous panuveitis frequently accompanied by neurological, auditory, and integumentary manifestations. It is considered to be an autoimmune condition triggered by several factors, including microbial infection. With the promotion of mass vaccination against SARS-CoV-2, cases of VKH disease associated with COVID-19 vaccines have emerged. Here, we have reported another case of probable VKH disease in close temporal association with the COVID-19 vaccination and have provided an overview of similar cases previously reported. We conclude that COVID-19 vaccines of different types are possible triggering factors of VKH disease with different manifestations. Nonetheless, all of the cases demonstrated a good response to steroid therapy. Although the causality is still unclear, physicians and ophthalmologists should be aware of this possible side effect and further clarify the clinical and epidemiological features, as well as the underlying mechanisms of it.

## Figures and Tables

**Table 1:** Naranjo adverse drug reaction (ADR) probability scale.

ADR Probability Scale					
To Assess the Adverse Drug Reaction, Please Answer the Following Questionnaire and Give the Pertinent Score					
		Yes	No	Do Not know	Score
1	Are there previous conclusive reports on this reaction?	+1	0	0	0
2	Did the adverse event appear after the suspected drug was administered?	+2	−1	0	2
3	Did the adverse reaction improve when the drug was discontinued or a specific antagonist was administered?	+1	0	0	0
4	Did the adverse reaction reappear when the drug was readministered?	+2	−1	0	0
5	Are there alternative causes (other than the drug) that could on their own have caused the reaction?	−1	+2	0	2
6	Did the reaction reappear when a placebo was given?	−1	+1	0	0
7	Was the drug detected in the blood (or other fluids) in concentrations known to be toxic?	+1	0	0	0
8	Was the reaction more severe when the dose was increased, or less severe when the dose was decreased?	+1	0	0	0
9	Did the patient have a similar reaction to the same or similar drug in any previous exposure	+1	0	0	0
10	Was the adverse event confirmed by any objective evidence?	+1	0	0	0
				Total score	4
Probability Category	<0: Doubtful	1–4: Possible 5–8: Probable >9: Definite			Possible

## Data Availability

The data that support the findings of this study, after adequate anonymization that protects the patient’s privacy, will be available on request from the corresponding authors (Q.C.). The data are not publicly available due to them containing information that could compromise research participant privacy/consent.
